# A Rare Case of Fungal Granuloma Confined to the Nasal Septum

**DOI:** 10.7759/cureus.65414

**Published:** 2024-07-26

**Authors:** Koichi Tamura, Kazuya Takeda, Takeshi Tsuda, Makiko Kawai, Hidenori Inohara

**Affiliations:** 1 Department of Otorhinolaryngology-Head and Neck Surgery, Osaka University Graduate School of Medicine, Suita, JPN; 2 Department of Pathology, Osaka University Graduate School of Medicine, Suita, JPN

**Keywords:** fungal infection, septal deviation, invasive fungal rhinosinusitis, nasal septum, granuloma

## Abstract

Granulomatous lesions in the nasal sinuses are associated with a variety of diseases, including immune disorders such as sarcoidosis, vasculitis, immunoglobulin G4 (IgG4)-related diseases, malignant lymphomas, and microbial infections. Here, we report a rare case of fungal granuloma that occurred exclusively within the nasal septum.

The patient presented to the Department of Surgery with the chief complaint of nasal obstruction associated with nasal septal deviation. A bulge was found below the right nasal septum. Initially, it was diagnosed as mucosal swelling associated with rhinitis, and surgery was performed. A granulomatous lesion with bone destruction was found under the mucosa of the nasal septum, which led to the diagnosis of fungal granulation based on postoperative pathology. Though bacterial and fungal infections of the nasal septum are occasionally observed, this is the first reported instance of a fungal granuloma confined to the nasal septum. Infection within the nasal septum, although rare, should also be considered as a differential diagnosis for morphological abnormalities of the nasal septum.

## Introduction

Granulomatous lesions develop as a result of chronic inflammation in the affected area and are commonly caused by an abnormal immune response or infection [1]. Granulomas, particularly those in the nasal sinuses, can result from diverse etiologies, including autoimmune diseases, lymphoproliferative diseases, and infections [2-7]. These lesions usually manifest on the mucosal surface of the nasal cavity and are diagnosed through clinical observation and pathological examination [1]. This report presents a rare case of a fungal granuloma confined to the submucosa of the nasal septum, which was incidentally detected in a patient who underwent surgery for nasal obstruction. This is an unusual case of an infection affecting only a specific area of the nasal septum, further illustrating the complexity of the diagnosis and treatment of sinonasal disease.

## Case presentation

**Figure 1:**
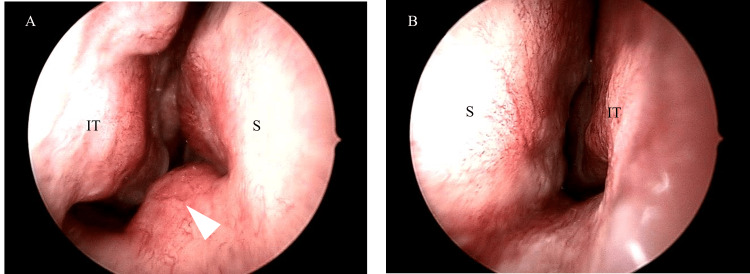
Initial physical examination (A) Right side, (B) Left side; Mucosal swelling below the right nasal septum (white arrowhead). IT: Inferior turbinate, S: Septum

The patient was a 50-year-old housewife who presented with nasal obstruction on both sides without pain or epistaxis and was diagnosed with nasal septal deviation and allergic rhinitis. She was referred to our department for surgical treatment because her symptoms persisted despite conservative treatment including nasal corticosteroids. A left convex nasal septal deviation and a bulge below the right nasal septum were observed (Figure 1). However, there was no abnormality on the mucosal surface, and we diagnosed it as normal mucosal swelling.

**Figure 2:**
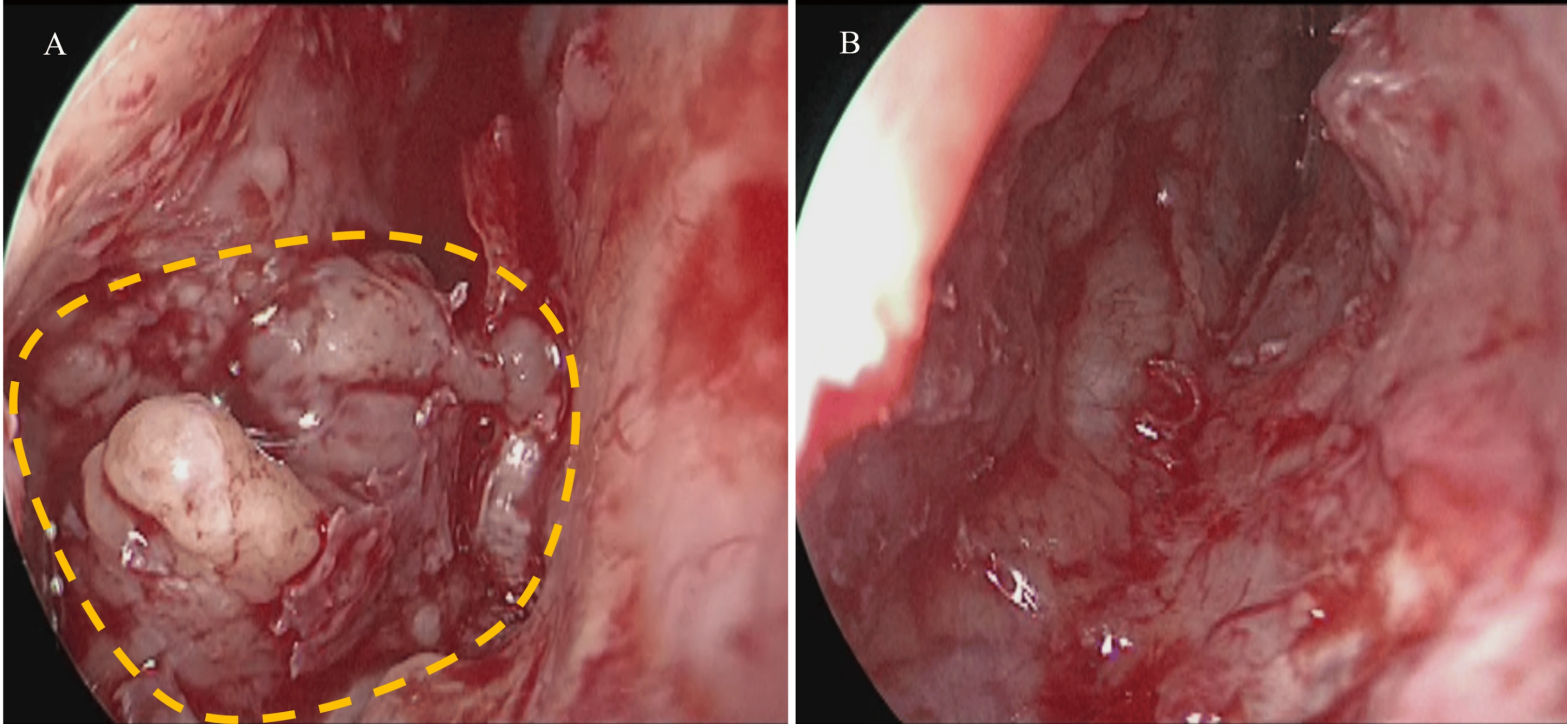
Intraoperative findings (A) Granulomatous lesions (indicated by the yellow line) with partial destruction of the vomer are observed within the nasal septum, (B) After resection

The patient underwent endoscopic septoplasty as planned. A Killian incision was made in the mucosa of the left nasal septum, and mucous membrane dissection revealed a granulomatous lesion with partial destruction of the vomer. The lesion was a soft granulomatous mass with indistinct borders but was localized within the nasal septum (Figure 2).

**Figure 3:**
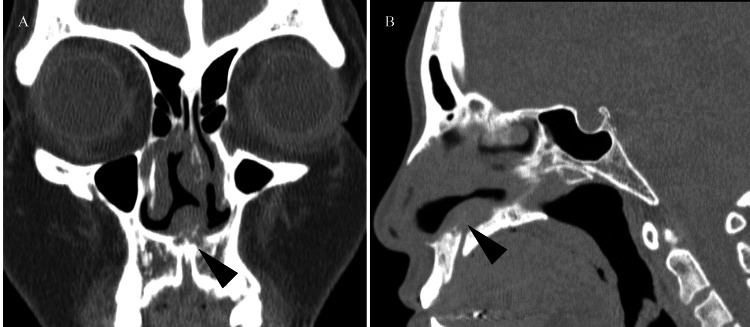
Preoperative CT scan. (A) Coronal. (B) Sagittal. Bone destruction is observed from the maxillary nasal ridge to the anterior part of the vomer (black arrowhead).

The intraoperative frozen section diagnosis revealed inflammatory granulation tissue; however, the precise cause, including infectious or vasculitic origin, was difficult to determine. The lesion was removed as much as possible, and the surgery was completed. A postoperative review of the preoperative CT scan revealed slight maxillary bone destruction (Figure 3).

**Figure 4:**
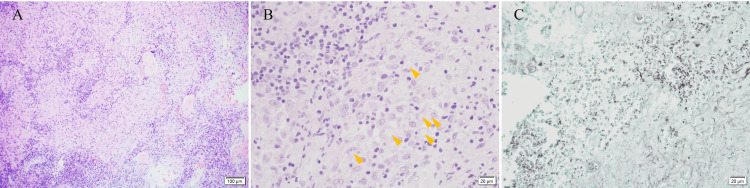
Histopathological findings (A) H&E staining (low-power field). Granulomatous lesions are present. (B) H&E staining (high-power field). Aggregation of epithelioid histiocytes and colorless areas (yellow arrowhead), which may be a fungus, are present. (C) Grocott staining. Yeast-like fungi congregate.

Blood tests were performed for ANCA-related vasculitis, IgG4-related disease, sarcoidosis, malignant lymphoma, and acid-fast infection; however, no obvious abnormalities were detected. A histopathological examination revealed a suspected fungal infection. Grocott staining confirmed the presence of yeast-like fungi, suspected to be Cryptococcus (Figure 4). However, no fungi were detected in culture tests, and 18S rRNA-seq using RNA extracted from formalin-fixed paraffin-embedded (FFPE) specimens failed to identify the fungal species. There was no history of surgery, trauma, or drug abuse, and no factors associated with susceptibility to infection such as diabetes, HIV infection, or the use of immunosuppressive drugs. The lesion was surgically removed as much as possible, and the patient was observed without treatment, with no apparent recurrence so far one year after surgery.

## Discussion

This case represents an exceptionally rare instance in which a preoperative diagnosis of mucosal swelling due to rhinitis led to the intraoperative detection of a granulomatous lesion within the nasal septum, which was subsequently identified as a fungal infection through postoperative histopathology. Granulomatous lesions in the nasal sinuses are occasionally encountered and can arise from various etiologies, including autoimmune diseases, lymphoproliferative disorders, and infections [2]. Typically, these lesions are manifested on the mucosal surface of the nasal cavity and are diagnosed through clinical observation, pathological examination, and culture tests. However, lesions confined to the submucosa, as in the present case, pose a significant diagnostic challenge [8]. The patient presented with nasal obstruction secondary to septal deviation. Initially diagnosed as mucosal swelling associated with rhinitis, a granulomatous lesion was incidentally detected during endoscopic septoplasty, revealing bone destruction of the vomer. Subsequent histopathological examination suggested fungal infection.

A nasal septal abscess, often associated with trauma or surgery, is a differential diagnosis for mucosal swelling of the nasal septum. In abscesses, the mucosal surface is often normal; however, the swelling is not confined to the lower part of the nasal septum, as seen in the present case. A few cases of fungal nasal septal abscesses have been reported [9], occurring in immunocompromised patients and following surgery for fungal rhinosinusitis. Although there have been no reports of spontaneous onset in healthy individuals, as in the present case, fungal infection should be considered when identifying submucosal lesions in immunocompromised individuals or after surgery for fungal rhinosinusitis.

To differentiate granulomatous lesions, blood tests were conducted to rule out ANCA-associated vasculitis, IgG4-related diseases, sarcoidosis, malignant lymphoma, and acid-fast infections. All results were negative, complicating the diagnosis. ANCA measurement aids in diagnosing vasculitis-related granulomas, whereas ACE and sIL-2R measurements help differentiate sarcoidosis from malignant lymphomas [3,5,7]. For suspected fungal infections, β-D glucan and each fungal antigen, including Aspergillus antigen, Candida antigen, and Cryptococcus antigen, measurements are valuable, though all were negative in this case.

The patient’s histopathology suggested Cryptococcus infection, but culture tests and 18S rRNA sequencing using RNA from FFPE specimens failed to identify the fungal species. Culture tests for fungi have poor sensitivity, and reports indicate that 18S rRNA sequencing can be effective [10]. However, the majority of these studies were based on fresh specimens. In this study, RNA extracted from FFPE specimens was used, and it is believed that quality deterioration due to RNA fragmentation affected the results. If the granulomatous lesion itself had been submitted for culture testing or if rapid RNA extraction had been performed during surgery, a definitive diagnosis might have been made.

Most cases of fungal rhinosinusitis are noninvasive; however, infections accompanied by granulomatous lesions with bone destruction, as seen in this case, are classified as invasive fungal rhinosinusitis [11]. Granulomatous invasive fungal rhinosinusitis, compared to other types of invasive fungal infections, often occurs in individuals with normal immune status and should be distinguished from other invasive types. Except for those that extend into the orbital or cranial cavity, the prognosis is generally good [11]. Although invasive fungal infections involving the nasal septum are frequently reported, cases confined exclusively to the nasal septum have not been reported.

Regarding the imaging assessment of granulomatous invasive fungal rhinosinusitis, MRI plays an important role in assessing the extent of the lesion and its involvement in the surrounding structures. First, on T1-weighted images, lesions usually appear as isosignals to low signals. On T2-weighted images, low-signal areas may be observed, suggesting a fungal component, calcification, or necrotic tissue, suggesting dense granulomatous tissue. In addition, surrounding inflammation or edema may appear as high signals [12]. In this case, MRI was not performed because the possibility of fungal infection was not considered preoperatively; however, it could have led to a diagnosis if it had been performed preoperatively.

Extensive debridement and antifungal therapy are the primary treatments for invasive fungal rhinosinusitis [11]. In this case, although identification of the fungal species was unsuccessful, surgical removal of the lesion was as comprehensive as possible. Given the patient’s immunocompetent status and the absence of typical risk factors for fungal infections (e.g., diabetes and HIV), postoperative management involved rigorous observation without the use of antifungal drugs. No recurrence was observed over one year of follow-up.

This case highlights the importance of considering fungal infections in the differential diagnosis of nasal septal morphological abnormalities, even in immunocompetent patients without predisposing factors. It also emphasizes the importance of submitting granulomatous lesions for culture tests and the potential utility of rapid RNA extraction during surgery for an accurate diagnosis.

## Conclusions

This case represents the first reported instance of a fungal granuloma confined exclusively to the nasal septum in an immunocompetent patient. It highlights the challenges in diagnosis and management. The case illustrates the importance of culture testing and the potential advantages of prompt RNA extraction from granulomatous lesions for accurate diagnosis. It also underscores the need to consider fungal infections in the differential diagnosis of nasal septal abnormalities. Enhanced awareness and thorough diagnostic evaluations are crucial for proper diagnosis and effective treatment in similar cases.
